# Optimizing Fuel‐Based Environmental Strategies for Stack Emissions From Zigzag and Fixed Chimney Bull’s Trench Kiln (FCBTK) Brick Kilns: A Comparative Analysis

**DOI:** 10.1155/ianc/9995698

**Published:** 2026-02-04

**Authors:** Sohaib Hasnain, Khalid Mahmood Zia, Nadia Akram, Toheed Asghar, Ayesha Tariq

**Affiliations:** ^1^ Department of Chemistry, Government College University Faisalabad, Faisalabad, 38030, Pakistan; ^2^ Environmental Lab/EPA Labs, Pakistan Environmental Protection Agency, Islamabad Faisalabad Campus House No. 277 Street No. 02 Mustafaabad, Faisalabad, 38040, Pakistan; ^3^ Department of Chemistry, University of Agriculture Faisalabad, Faisalabad, 38040, Pakistan, uaf.edu.pk

**Keywords:** Air pollution, brick kiln, coal, renewable fuels, stack emission

## Abstract

Pakistan has around 20,000 brick kilns, constituting 3% of global brick production. Consequently, air pollution and air quality indicators have significantly deteriorated. In this study, we examined the effect of different fuel types, such as coal, coal + wheat straw, and coal + rice straw, on stack emissions from FCBTK kilns and Zigzag kilns in Faisalabad, Pakistan. Standard protocols for measuring stack emissions, including smoke opacity, SO_2_, and CO, were used. The results were compared with Pakistan’s Punjab Environmental Quality Standards (PEQS). Specifically, in Zigzag kilns, blended fuels reduced CO emissions to approximately 123.7 mg/Nm^3^ with wheat straw and 162.2 mg/Nm^3^ with rice straw. In contrast, FCBTKs showed CO emission reductions of 233.17 mg/Nm^3^ with wheat straw and 341.07 mg/Nm^3^ with rice straw. For SO_2_, Zigzag kilns achieved reductions of 412.7 mg/Nm^3^ with wheat straw and 352.4 mg/Nm^3^ with rice straw, while FCBTKs reduced emissions by 564.9 and 481.7 mg/Nm^3^, respectively. Smoke opacity in Zigzag kilns improved by 4.6 percentage points with rice straw and 2.3 with wheat straw, whereas in FCBTKs, the improvement was 41.4 and 20.3 percentage points, respectively. Statistical comparisons using Pearson’s correlation and linear regression analysis further indicate that biomass additives combined with coal are more effective as fuels in the brick kiln industry. One‐way ANOVA and Kruskal–Wallis tests, along with post hoc analysis, confirmed significant differences among fuel groups (*p* < 0.001), demonstrating strong relationships between fuel composition and emission levels. These additives significantly reduce air pollution and improve community health by lowering emissions of smoke opacity, SO_2_, and CO. According to the results, blending agricultural residues with coal enhances emission performance, with Zigzag kilns showing the most significant reductions.

## 1. Introduction

In the 21st century, air pollution has become one of the most serious environmental concerns, and its impacts have grown increasingly evident [1]. It has affected human health as well as living standards over the years, particularly in recent decades [2]. The health problems resulting from air pollution are among the major threats to people today [3]. Brick production contributes significantly to traditional air pollution [4]. Most Asian brick kilns are owned by low‐income individuals and produce approximately 140 billion bricks annually [5]. Due to coal burning, these brick kilns emit large amounts of SO_2_ and particulate matter (PM), which cause respiratory problems and degrade air quality [6]. Brick‐making in Pakistan is a rapidly growing industry that accounts for nearly 3% of the world’s brick production [7]. With Pakistan’s increasing population, brick production and demand have also continued to rise [8]. Approximately 20,000 brick kilns operate across Pakistan, located in both rural and urban areas [9].

During recent years (2019–2023), various government and international organizations have collaborated to promote greener and more energy‐efficient brick kiln technologies [10]. Brickmakers now utilize vertical shaft brick kilns (VSBKs), tunnel kilns, and Hoffmann kilns to manufacture bricks [11]. Zigzag kilns are preferred in Pakistan and other South Asian countries because they are more energy‐efficient and environmentally friendly than fixed chimney Bull’s trench kilns (FCBTKs) [12]. Currently, Pakistan primarily employs two types of kilns [13]. Traditional brick kilns (FCBTKs) are fueled by coal and ground rubber [14]. Zigzag technology has been introduced in recent years to replace conventional FCBTKs [11]. With this technology, hot air moves in a Zigzag pattern, transferring heat from the gases to the bricks, thereby reducing black carbon emissions by more than 60% compared to traditional brick kilns [11]. Kilns with Zigzag firing consume 20% less fuel, produce up to 70% lower emissions, and are significantly less harmful to the environment [15].

Bricks are produced from clay or river sediments containing fine particles [16]. Traditional brick kiln fuels include Assam coal, slack coal, and lignite, which contain very high levels of sulfur and ash (25%–30%) [17]. In brick kilns, coal is the primary fuel source, followed by sawdust, wood, and other wood‐processing by‐products [18]. The combustion of coal produces significant amounts of sulfur dioxide and black carbon [19]. Many low‐grade carbonaceous fuels serve as potential alternatives to coal, such as rice straw, bagasse, and wood or sawdust [20]. In the Indian region alone, there are approximately 140,000 small‐ and medium‐sized brick kilns that burn between 4 and 5 million tons of coal annually [21]. Researchers estimate that over 1 trillion bricks are produced each year and that approximately 110 million tons of fossil fuels are consumed annually [22]. Brick manufacturing negatively impacts human health, ecosystem quality, climate change, and resource depletion [23]. It has been proposed that adopting cleaner fuels in modern kilns could significantly reduce these emissions; however, there is limited empirical evidence evaluating this integrated approach under field conditions [11].

In the present study, we address a gap in existing research regarding how fuel choices influence emissions across different kiln types. The general benefits of Zigzag kilns over traditional FCBTKs are known, but their specific impacts (e.g., blending coal with biomass) in each kiln type remain largely unknown. Our study addresses this gap by comparing stack emissions from traditional FCBTKs and modern Zigzag kilns under three fuel scenarios: coal alone, coal mixed with wheat straw, and coal mixed with rice straw. Compared to previous studies in brick kiln research, this fuel‐based emissions analysis and comparative methodology provide a new perspective on how fuel selection and kiln design affect air pollution.

In this work, we measured key pollutants including smoke opacity, SO_2_, and carbon monoxide (CO) emitted from each kiln type under the different fuel combinations. Standard emission monitoring protocols were followed to ensure reliable and comparable results. We further applied statistical analyses (Pearson’s correlation, linear regression, one‐way ANOVA, Kruskal–Wallis tests, and post hoc analysis) to evaluate the relationships between fuel composition and emission levels. The results of our study add new evidence to environmental science as well as to kiln emission management by identifying which fuel–kiln combinations reduce harmful emissions most effectively. Moreover, the findings demonstrate empirically that combining cleaner biomass fuels with improved kiln technologies can reduce air pollutants substantially, supporting theoretical expectations for sustainable pollution control. A major benefit of this research is that it not only fills a research gap but also provides practical guidance for policymakers and industry stakeholders in developing targeted environmental strategies to curb brick manufacturing emissions.

## 2. Material and Method

### 2.1. Site Description

**FIGURE 1:**
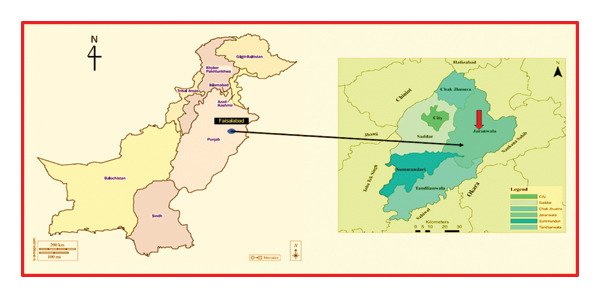
Location of the study area in Faisalabad, Pakistan.

Regarding population size, Pakistan is the sixth most populous country in the world, with a population of 212.82 million people. Geographically, it lies between 60°50′ to 77°50′ East and 23°35′ to 37°05′ North. Its landscape is diverse, bordered by the Arabian Sea, Iran, India, the Himalayas, Afghanistan, and China. Pakistan has three main regions: the Baluchistan Plateau in the west and south, the Indus River basin plain in the center and east (covering 65% of the total area, approximately 796,096 km^2^), and the northern highlands, which include the Himalayas, Karakoram range, and the Hindu Kush [24]. There are approximately 20,000 FCBTK brick kilns in Pakistan, with 10,347 of them located in Punjab [25]. Currently, approximately 10,000 brick kilns in Punjab have transitioned to Zigzag technology, which is designed to minimize emissions and enhance fuel efficiency. Notably, about 3.1 million workers are employed in the brick manufacturing industry in Punjab [26]. A study was conducted between March 26 and April 24, 2023, to measure brick kiln emissions in Tehsil Jaranwala, District Faisalabad, Punjab, Pakistan, as illustrated in Figure 1. The study area, Jaranwala, is located at 31°20′ North latitude and 73°26′ East longitude, situated between two canals, the Rakh Branch (RB) and Gogera Branch (GB). It covers an area of 437,386 acres (1777.04 km^2^) and is 35 km southeast of Faisalabad, accessible via the Lahore–Faisalabad road and Jaranwala–Khurrianwala road [27]. The brick industry in Punjab follows an old‐fashioned seasonal methodology that begins during the winter [28]. Currently, most kilns are being converted to Zigzag kilns; however, many FCBTKs remain operational. During the study, a total of 30 FCBTK kilns and 30 Zigzag kilns with varying fuel consumption proportions were examined in Tehsil Samundari, District Faisalabad.

### 2.2. Overview of Brick Kilns in Pakistan

**FIGURE 2:**
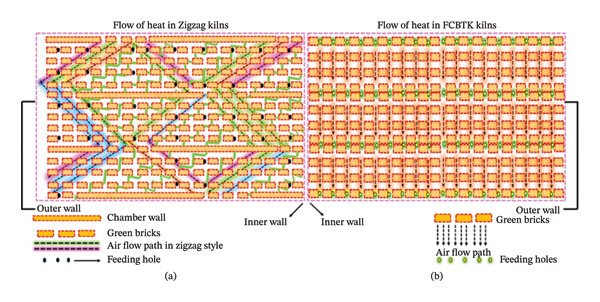
Heat transfer mechanism in Zigzag and FCBTK kilns. (a) Flow of heat in Zigzag kilns. (b) Flow of heat in FCBTK kilns.

There are two broad categories of brick kilns in Pakistan: (1) FCBTKs, which are traditional structures, and (2) modern improved Zigzag kilns, as illustrated in Figure 2. In FCBTKs, the fire travels directly through the gaps formed by green (unbaked) bricks, which are stacked between the outer and inner kiln walls. Once the green bricks are fired, they are moved to the front of the firing zone, while the fired bricks are removed from the rear (cooling zone). Natural‐draught kilns use a chimney to generate the required draught for combustion. As cold air enters the cooling zone through the gaps created by the stacked fired bricks, the temperature of the fired bricks decreases, while the temperature of the air entering the firing zone rises. Heat is transferred from the heating zone to the firing zone, supplying the excess air required for combustion, which then moves upward through the chimney duct (Figure 2).

A Zigzag kiln also involves stacking green bricks to create passages for fire flow. During combustion, a fan‐driven draught system is employed. Zigzag kilns are similar to FCBTKs but differ primarily in two aspects: (1) the stacking pattern of the unbaked bricks and (2) the rectangular layout of the outer and inner walls instead of oval shapes. In both kiln types, the cooling, firing, and preheating zones share similar functional roles. However, in Zigzag kilns, the elongated and angled fire path ensures steady heat transfer to the bricks, resulting in improved combustion efficiency and lower fuel consumption. Furthermore, modern Zigzag kilns play a key role in capturing PM along the kiln walls. Because coal is fed in smaller, controlled quantities and the flue gases are repeatedly diverted by the Zigzag flow path, a turbulent airflow is created that enhances combustion and promotes the effective removal of wall‐borne particulates. This process leads to reduced fuel consumption and emissions, providing substantial environmental benefits. By emphasizing these advantages, the study highlights the urgent need to transition toward more sustainable brick kiln technologies in Pakistan.

### 2.3. Stack Sampling and Instrumentation

Chimneys of brick kilns in Pakistan are typically 20–30 m high. According to the standards set by the Bureau of Indian Standards (BIS) and the United States Environmental Protection Agency (US EPA), stack sampling ports were installed at heights between 15 and 20 m above ground level, each with a diameter of approximately 10 cm. Carbon monoxide (CO) and sulfur dioxide (SO_2_) emissions were measured using a portable HORIBA PG‐250 flue gas analyzer. The device employs chemiluminescence for NOx determination and nondispersive infrared absorption (NDIR) for the measurement of SO_2_, CO_2_, and CO, ensuring high accuracy and precision. The HORIBA PG‐250 provides several detection ranges (SO_2_: 0–3000 ppm; CO: 0–5000 ppm) with linearity within ±2.0% of full scale (F.S.) and a sample gas flow rate of approximately 0.4 standard liters per minute (SLPM).

The analyzer was routinely calibrated using certified zero and span gases to maintain optimal measurement accuracy. Zero calibration was performed before daily sampling, while weekly span checks were conducted to verify consistency. The analyzer probe was securely fitted into the chimney’s sampling port to ensure an airtight connection and prevent ambient air contamination. Gas samples were continuously drawn into the analyzer during each sampling interval, providing real‐time concentration data that were digitally recorded for subsequent analysis [29, 30]. Smoke opacity was assessed using the Ringelmann chart. Real‐time and integrated sample readings were averaged to generate the values. The measurement parameters, their underlying principles, and the reference methods used are summarized in Table 1.

**TABLE 1:** Measurement parameters, reference methods, and underlying principles.

Parameters	Principle	Reference method
Oxides of sulfur	NDIR (Nondispersive infrared absorption)	USEPA method 6C
Oxides of carbon	NDIR (Nondispersive infrared absorption)	EN 15058 and USEPA method 10
Smoke opacity	(Ringelmann chart) Compare plume opacity according to opacity levels	Method 9 USEPA

### 2.4. Instrument Adaptation and Chimney Application

The HORIBA PG‐250 analyzer is specifically designed for stack gas measurements and can be directly inserted into chimney sampling ports (Figure 3). Its rugged and compact design ensures reliable field operation under varying environmental conditions. Only minor modifications were made, including securely mounting the analyzer probe within the stack ports and protecting sensitive electronic components from dust and moisture commonly present at brick kiln sites. This configuration enabled accurate, real‐time measurement of gas concentrations, which are essential for evaluating emission rates in accordance with established monitoring protocols.

FIGURE 3(a) HORIBA PG‐250 flue gas analyzer used for sampling kiln chimney gases; (b) smoke emissions from an FCBTK kiln; (c) smoke emissions from a Zigzag kiln.

**Figure:**
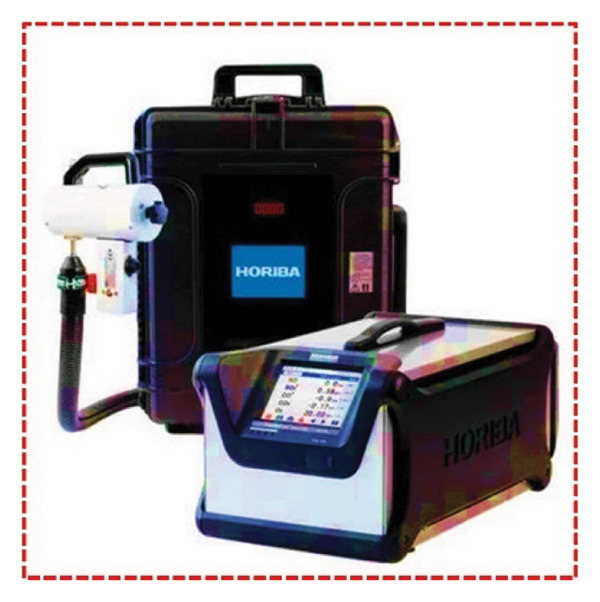
(a)

**Figure:**
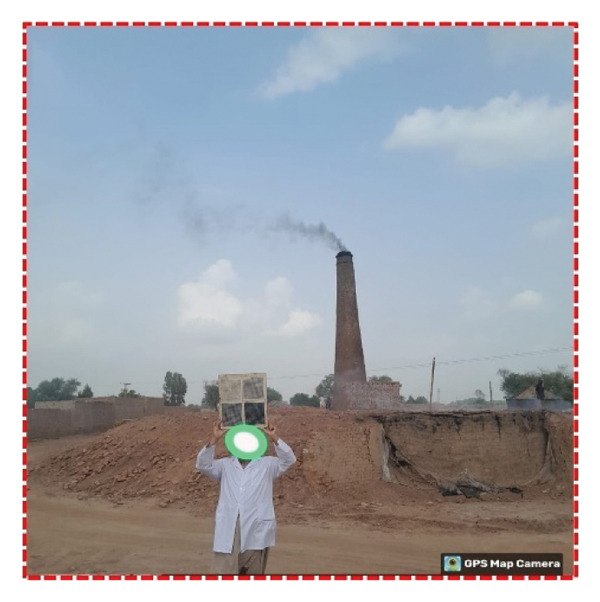
(b)

**Figure:**
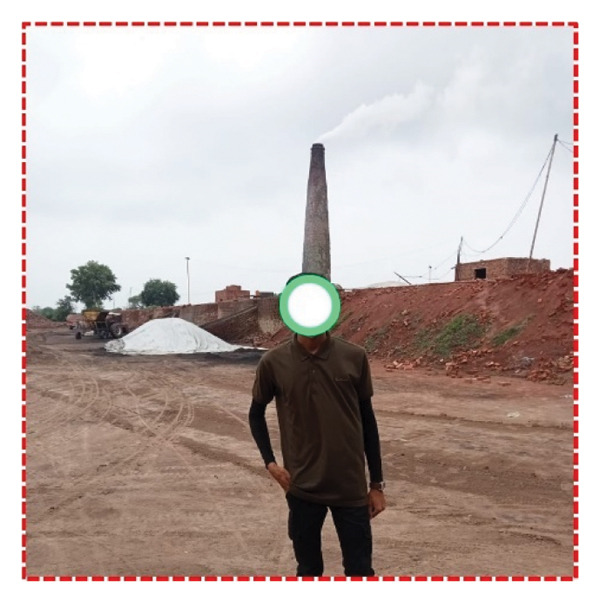
(c)

Results for pollutant gas and particulate concentrations were compared with the Pakistan Environmental Quality Standards for industrial gaseous emissions, which were notified by the Government of Pakistan (https://epd.punjab.gov.pk/peqs). These benchmark standards are presented in Table 2.

**TABLE 2:** Punjab (Pakistan) environmental quality standards (PEQS) for industrial gaseous emissions (mg/Nm^3^).

Parameters	Standards for industrial gaseous emissions (mg/Nm^3^)
Sulfur dioxide (SO_2_)	1700
Carbon monoxide (CO)	800
Particulate matter	500
Smoke opacity	40% by Ringelmann chart
Lead	50
Mercury	10

Groups of FCBTK and Zigzag brick kilns were analyzed with respect to pure and mixed fuel use. The different types of brick kilns and fuel combinations selected for the investigation, along with their respective fuel consumption and brick production data, are summarized in Table 3.

**TABLE 3:** Types of brick kilns and fuels selected for the investigation.

Types of kilns	Number of kilns (n)	Fuel used	Average fuel consumption (kg/day)	Average brick production per day
FCBTK	10	Coal	4600	25,000
FCBTK	10	90% coal + 10% wheat straw	4750	23,500
FCBTK	10	90% coal + 10% rice straw	4700	23,500
Zigzag	10	Coal	3250	21,000
Zigzag	10	90% coal + 10% wheat straw	3355	22,500
Zigzag	10	90% coal + 10% rice straw	3228	20,000

### 2.5. Statistical Analysis

Pearson’s correlation method was used to analyze the relationship between the selected fuel types, kiln types (FCBTK and Zigzag), and stack emissions (smoke opacity (%), CO, and SO_2_) using OriginPro 2024 software. Linear regression model, one‐way ANOVA, and Kruskal–Wallis tests were also employed to evaluate the impact of different fuel types on stack emissions, such as smoke opacity, CO, and SO_2_, in FCBTK and Zigzag kilns. The stack emissions (dependent variables), including smoke opacity (%), CO, and SO_2_, were plotted against three fuel types: coal (coded as 0), coal + wheat straw (coded as 1), and coal + rice straw (coded as 2).

For each fuel combination and kiln type, all types of emission measurements were taken in triplicate, with three independent readings recorded from each kiln under steady‐state operating conditions. These replicates were spaced with adequate time intervals to ensure statistical independence. The resulting data were averaged and reported as mean ± standard deviation to represent the variability within each group. The instrument was recalibrated before each measurement session to reduce systematic error, and environmental conditions (temperature and humidity) were checked to provide consistency between trials.

## 3. Results and Discussion

### 3.1. Smoke Opacity

Smoke is an odorless yet harmful byproduct generated during the combustion of fuel in brick kilns. The degree to which PM (smoke) obstructs visibility is referred to as smoke opacity. It measures the darkness of flue gases resulting from unburned carbon particles emitted during incomplete combustion. Ringelmann scales are used to quantify smoke opacity based on the intensity of its darkness. Generally, a higher opacity value indicates darker or denser smoke, whereas a lower opacity value represents clearer emissions [12]. PM enters the ambient air through various sources, including wildfires, burning wood and fossil fuels, vehicular emissions, brick kiln emissions, and industrial activities. So, it is critical to understand how brick manufacturing processes affect air quality and environmental impacts. In the present study, the density and opacity of smoke emissions from FCBTK and Zigzag kilns were determined using a smoke chart or Ringelmann chart. This chart comprises black and white rectangles or squares ranging from 0% (white) to 100% (black) opacity. The Ringelmann scale value describes the following conditions [31, 32]:

• A value of 0 indicates 0% density, and a background of 100% is proportional to it.
• A value of 1 indicates 20% density, and a background of 80% is proportional.
• A value of 2 indicates 40% density, and a background of 60% is proportional.
• A value of 3 indicates 60% density, and a background of 40% is proportional to it.
• A value of 4 indicates 80% density, and a background of 20% is proportional.
• To summarize, a value of 5 indicates 100% density, which means the color or shade is at its maximum intensity, and no background can be seen.

To evaluate smoke emissions’ opacity, the observer uses a standardized color chart. An observer typically selects an indicator from the chart corresponding to the observed opacity level, which is used to estimate the density of the smoke. Smoke emissions are dense and opaque, with opacity levels directly related to their environmental impacts [33]. Initially, this method was employed to assess agricultural machinery performance [34]; however, it has since been applied in air pollution management and monitoring.

Smoke opacity differences between kiln types indicate the potential for technological intervention to significantly reduce greenhouse gas emissions in traditional industries such as brick production [12]. In Table 4, we compare pure coal with 90% coal and 10% wheat straw, and 90% coal with 10% rice straw, which provides insight into how fuel composition impacts emissions. The measured smoke opacity values were 72.3 ± 4.90, 51.9 ± 5.34, and 30.8 ± 1.68 for FCBTK kilns, and 19.0 ± 0.94, 16.7 ± 0.67, and 14.4 ± 0.69 for Zigzag kilns using different fuel mixtures, highlighting the efficacy of Zigzag technology in reducing PM emissions, as shown in Figure 4(a).

**TABLE 4:** Emissions of smoke opacity (%) from different fuels in FCBTK and Zigzag kilns.

Fuel	FCBTK (%)	Zigzag (%)	PEQS
Coal	72.3 ± 4.90	19 ± 0.94	40% by Ringelmann chart
90% coal + 10% wheat straw	51.9 ± 5.34	16.7 ± 0.67	
90% coal + 10% rice straw	30.8 ± 1.68	14.4 ± 0.69	

FIGURE 4Measured concentrations of stack emissions: (a) Smoke opacity (%) for different fuels; (b) SO_2_ concentration (mg/Nm^3^) for different fuels; (c) CO concentration (mg/Nm^3^) for different fuels.

**Figure:**
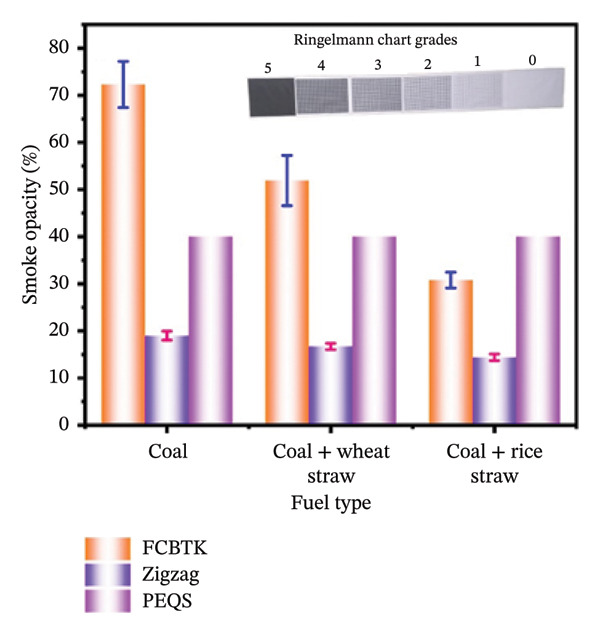
(a)

**Figure:**
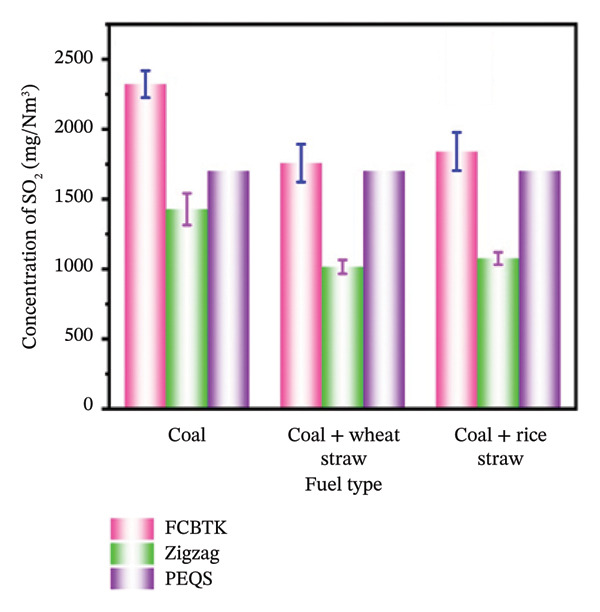
(b)

**Figure:**
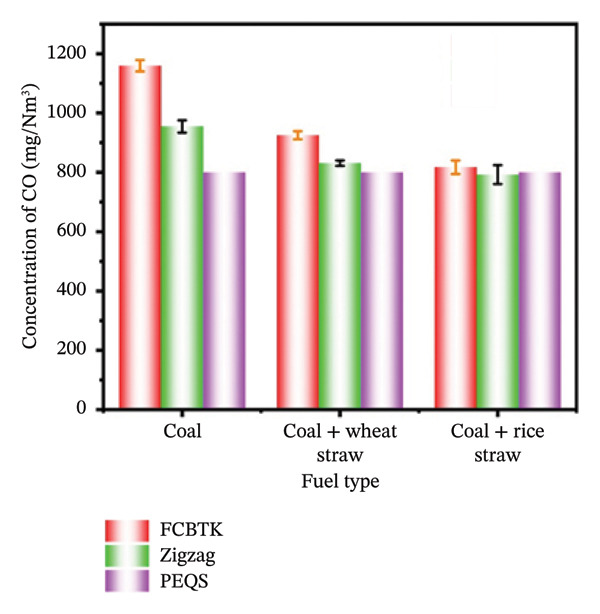
(c)

Reduced particulate emissions play a critical role in minimizing health risks associated with air pollution and its related harms, as demonstrated by these findings in the broader environmental management context. Based on the present study, smoke opacity measured from Zigzag kilns with a fuel charge is lower than that of the FCBTK and PEQS standards. Observations from Zigzag kilns showed a Ringelmann number of 1. By utilizing Zigzag kiln technology, brick manufacturers can reduce their environmental footprint and contribute to sustainable development goals. In FCBTK kilns, the average Ringelmann number was measured as 4 with pure coal, 3 when using a blend of 90% coal and 10% wheat straw, and 2 with a blend of 90% coal and 10% rice straw. In conclusion, our findings contribute valuable data to environmental management. Through fuel modification, agricultural residues can further reduce emissions by decreasing opacity, which could be a significant area for future research and policy development [35]. In addition to reducing air pollution, heating or firing the materials in a Zigzag pattern could be adopted by other pollution‐intensive industries, thus contributing to the global effort to reduce pollution [12]. Our study is in strong agreement with previous research on the performance of diesel engines using fuel mixtures. It was observed that the smoke opacity value decreased with variations in fuel type [36, 37]. The decreasing order of fuel types in smoke production is as follows:

(1) Coal>9010% coal+% wheat straw>9010% coal+% rice straw.

### 3.2. Sulfur Dioxide

Due to the pollutant’s role in acid rain, smog, and respiratory issues, it is critically important to reduce SO_2_ emissions [38]. It also severely damages crops, forests, buildings, and lakes. Pakistan’s brick kilns cause significant atmospheric pollution. The use of cleaner technologies and fuel mixtures could reduce air pollution in the brick manufacturing industry. PEQS for SO_2_ emissions indicate the need for regulatory compliance [39]. The sustainability and environmental effects of fuel mixtures depend on the fuel composition and their impact on emissions. The present study examines how fuel types affect sulfur dioxide (SO_2_) emissions from FCBTK and Zigzag brick kilns in Pakistan. In both kiln types (FCBTK and Zigzag), SO_2_ was measured using a flue gas analyzer. Figure 4(b) shows a comparison of the current study’s results with those of the PEQS. Moderate and high emission levels compared to PEQS standards were observed when using 90% coal + 10% wheat straw and 90% coal + 10% rice straw as fuels, respectively, in FCBTK and Zigzag kilns.

In the present comparative study, it was found that SO_2_ emissions differ based on kiln type and fuel usage, with mixed fuels reducing emissions significantly compared to pure coal. Specifically, FCBTK kilns produced average SO_2_ concentrations of 2322.3 ± 96.2 mg/Nm^3^ when fueled with coal, while the addition of 10% wheat straw and 10% rice straw led to reduced values of 1757.4 ± 135 mg/Nm^3^ and 1840.6 ± 136.6 mg/Nm^3^, respectively, as shown in Figure 3(b). Zigzag kilns exhibited even lower SO_2_ emissions, measured at 1427.7 ± 114 mg/Nm^3^ for coal, 1015.0 ± 49.5 mg/Nm^3^ for the wheat straw blend, and 1075.3 ± 44.2 mg/Nm^3^ for the rice straw blend (Figure 3(b)). The results in Table 5 also show that FCBTK produces a higher pollutant concentration when using coal as fuel compared to Zigzag kilns. Therefore, shifting FCBTK kilns to more sustainable technologies and fuels is necessary, as their emissions exceed PEQS limits, especially when using pure coal.

**TABLE 5:** Emission of SO_2_ (mg/Nm^3^) for different fuels in FCBTK and Zigzag kilns.

Fuel	FCBTK (mg/Nm^3^)	Zigzag (mg/Nm^3^)	PEQS (mg/Nm^3^)
Coal	2322.3 ± 96.21	1427.7 ± 114	1700
90% coal + 10% wheat straw	1757.4 ± 135	1015 ± 49.45	
90% coal + 10% rice straw	1840.6 ± 136.6	1075.3 ± 44.15	

On the other hand, it also indicates that Zigzag kilns are better at minimizing SO_2_ emissions, particularly when coal is combined with renewable fuels such as wheat straw and rice straw. SO_2_ emissions from Zigzag kilns were also lower compared to PEQS. Therefore, in environmental management, adopting cleaner brick kiln technologies is essential globally due to the consistency of environmental conditions across different geographical contexts [40, 41]. Due to its higher sulfur content, rice straw emits more SO_2_ when added to coal. This suggests that Zigzag kilns and mixed fuels are effective methods for reducing SO_2_ emissions and achieving cleaner air [17].

Our findings agree with those of regional studies conducted in Pakistan (Peshawar), Nepal, and Bangladesh. In a study conducted in Peshawar, the utilization of pure coal and mixed fuels in different seasons was applied to examine the concentration of SO_2_, and values of 82.61, 75.85, 88.42, and 73.89 μg/m^3^ were measured [13]. Similarly, a study in Nepal found average concentrations of SO_2_ 735 ± 728 mg/m^−3^, and 371 ± 299 mg/m^−3^ from FCBTK and Zigzag, respectively [17]. A similar study in Bangladesh reported average SO_2_ concentrations of 332 ± 196 mg/m^3^ and 578 ± 354 mg/m^3^ from Zigzag and FCBTK kilns, respectively [30]. As mentioned above, the continuous emission from brick kilns harms human health and the environment. So, studies have shown that mixed fuel contributes significantly to its control. When (90% coal + 10% rice straw) is utilized as a fuel, a slightly higher value of SO_2_ was observed due to the higher sulfur contents in rice straw than wheat straw. The decreasing order of fuel type in the production of pollutants is as follows:

(2) Coal>9010% coal+% rice straw>9010% coal+% wheat straw.

### 3.3. Carbon Monoxide

Carbon monoxide (CO) impacts air quality, and its contribution to ground‐level ozone formation increases global warming and poses serious health risks, particularly respiratory discomfort [42]. CO damages human health and the environment [43]. Therefore, it is important to distinguish CO emissions from two types of kilns, namely, FCBTK and Zigzag kilns. Generally, FCBTK kilns produce more CO emissions than Zigzag kilns. Zigzag kilns produce fewer carbon monoxide (CO) emissions due to their more efficient fuel‐burning ability. This study examined how fuel types affect CO emissions from FCBTK and Zigzag brick kilns in Pakistan. Table 6 shows the mean concentrations of CO emissions from FCBTK kilns as 1159.6 ± 19.1, 925 ± 13.65, and 817.1 ± 22.83 mg/Nm^3^ for coal, coal + wheat straw, and coal + rice straw, respectively. These values are considered high and contribute significantly to air pollution. In the case of Zigzag kilns, the mean values were 954.6 ± 20.82, 830.9 ± 9.3, and 792.4 ± 31.88 mg/Nm^3^ for coal, coal + wheat straw, and coal + rice straw, respectively. The results indicate that Zigzag kilns are more promising in reducing CO emissions. This is due to more complete fuel combustion and the conversion of CO into CO_2_ in Zigzag kilns as opposed to FCBTK [30].

**TABLE 6:** Emission of CO (mg/Nm^3^) for different fuels in FCBTK and Zigzag kilns.

Fuel	FCBTK (mg/Nm^3^)	Zigzag (mg/Nm^3^)	PEQS (mg/Nm^3^)
Coal	1159.6 ± 19.1	954.6 ± 20.82	800
90% coal + 10% wheat straw	925 ± 13.65	830.9 ± 9.3	
90% coal + 10% rice straw	817.1 ± 22.83	792.4 ± 31.88	

The current study results become more relevant when considering Pakistan’s environmental regulations. Figure 4(c) illustrates the data obtained from FCBTK and Zigzag kilns using different fuels, compared with Pakistan’s PEQS. Pakistan’s PEQS set the limit for CO emissions at 800 mg/Nm^3^. Based on the results, it was observed that CO emission levels were much higher than the recommended standards in FCBTK, while Zigzag kiln emissions were slightly above or below the standard. This highlights the need for more precise environmental regulations and policies. The analysis reveals that CO emission levels are greatly affected by the type of fuel used. Coal‐fired kilns generate the highest levels of emissions [29].

In contrast, biobased renewable fuels, such as wheat and rice straw, yield much lower emissions. Biobased renewable agricultural waste blended with traditional coal as fuel could effectively reduce emissions. Therefore, in the context of environmental management, to control air pollution and health hazards associated with CO emissions, scientists should collaborate to promote Zigzag kiln technology and cleaner fuels [11]. The current study is in strong agreement with previous research conducted in the Asian region, such as in Bangladesh. A study carried out in Bangladesh showed that the average concentrations of CO were 177 ± 81 mg/m^3^ and 264 ± 75 mg/m^3^ from Zigzag and FCBTK kilns, respectively. The lower values, compared with the current study, might be attributed to the type and composition of fuel [30]. Using fuel (90% coal + 10% wheat straw), the higher CO value was reported owing to the greater carbon content in wheat straw than in rice straw. The higher CO concentration in wheat straw as fuel is due to its higher carbon content than rice straw [44]. In the context of the current study, the following fuels are ranked from high to low in terms of CO emissions:

(3) Coal>9010% coal+% wheat straw>9010% coal+% rice straw.

### 3.4. Comparative Study of Impact of Adopted Mitigation Technology

The effects of high exposure to brick kiln emissions on air quality and health cannot be downplayed, as it directly contributes to deteriorated public health. This study investigates how different fuel types influence emissions (smoke opacity fraction (%), SO_2_ (mg/Nm^3^), and CO (mg/Nm^3^)) from traditional FCBTK kilns and modern Zigzag kilns in Pakistan. Most brick kilns in the Faisalabad region use FCBTK technology, operating mainly in winter by burning coal or coal mixed with agricultural residues such as rice or wheat straw after the harvest season. Table 7 presents a comparative analysis of gaseous pollutant levels from selected Zigzag and FCBTK kilns, along with stack emission data from previous relevant studies conducted in neighboring countries.

**TABLE 7:** Comparative analysis of emissions from brick kiln types and fuel compositions across different studies.

Study	Location	Kiln type	Fuel composition	SO_2_ emission (mg/Nm^3^)	CO emission (mg/Nm^3^)	Smoke opacity (%)	Key findings
Current Study	Pakistan	FCBTK	Coal	2322.3 ± 96.21	1159.6 ± 19.1	72.3 ± 4.90	Higher emissions with coal; blending fuels reduces SO_2_, CO, and smoke opacity
		FCBTK	90% Coal + 10% Wheat Straw	1757.4 ± 135	925 ± 13.65	51.9 ± 5.34	Significant reduction in all pollutants with wheat straw mix
		Zigzag	Coal	1427.7 ± 114	954.6 ± 20.82	19 ± 0.94	Zigzag kiln has better combustion efficiency and lower emissions
		Zigzag	90% Coal + 10% Rice Straw	1075.3 ± 44.15	792.4 ± 31.88	14.4 ± 0.69	Zigzag with mixed fuels shows lowest emissions and opacity values.

Nepal Study	Nepal	FCBTK	Coal	735 ± 728	—	—	Regional variation in emissions due to fuel type and kiln design [17].

Bangladesh Study	Bangladesh	Zigzag	Coal	332 ± 196	177 ± 81	—	Similar results for CO reduction in Zigzag kilns; highlights regional adoption of cleaner technologies [30].

### 3.5. Statistical Correlation Analysis

In this study, Pearson’s correlation coefficient (r) was used to assess the relationships between kiln type, fuel composition, and measured emissions such as smoke opacity, carbon monoxide (CO), and sulfur dioxide (SO_2_). The Pearson correlation coefficient (r) indicates the strength and direction of a linear relationship between two variables, with values close to +1 or −1 representing a strong positive or negative relationship, respectively, and values near 0 indicating a weak or no correlation. In environmental studies, *r* values above 0.7 (positive or negative) are commonly considered strong [45], while values between 0.3 and 0.7 are considered moderate [46]. The level of significance was set at *p* < 0.05, indicating that the observed correlation is unlikely to be due to random chance [47]. Table 8 summarizes the Pearson correlation results for key variable pairs in both kiln types, alongside their significance levels and supporting literature references. There was a strong negative correlation, which was observed between fuel type and smoke opacity in both kiln types (*r* = −0.98 for FCBTK and *r* = −0.93 for Zigzag, both *p* < 0.001), meaning that increasing the use of biomass in the fuel mix strongly reduces visible emissions. Similarly, there was a strong positive correlation between CO and smoke opacity in Zigzag kilns (*r* = 0.96, *p* < 0.001), indicating that higher CO emissions are associated with denser smoke, consistent with the literature on incomplete combustion [50]. The positive correlations between SO_2_ and CO (*r* = 0.83 for FCBTK, *r* = 0.93 for Zigzag) suggest that conditions that increase one pollutant tend to increase the other as well, possibly indicating less efficient combustion. These strong, statistically significant relationships confirm that both fuel choice and kiln design are key drivers of emission reductions, supporting findings from recent studies on brick kiln pollution and clean fuel strategies [51, 52]. Using more biomass‐based fuels and modern kiln technology effectively reduces harmful emissions, with clear and credible statistical support [53].

**TABLE 8:** Pearson correlation results for the main variable pairs across both kiln types, along with significance and literature references supporting their interpretation.

Relationship examined	Kiln type	Pearson (*r* value)	Significance (*p*‐value)	Strength of correlation	Supporting reference
Fuel type vs. smoke opacity	FCBTK	−0.98	< 0.001	Strong negative	[48]
Fuel type vs. smoke opacity	Zigzag	−0.93	< 0.001	Strong negative	[48]
CO vs. smoke opacity	Zigzag	0.96	< 0.001	Strong positive	[33]
SO_2_ vs. CO	FCBTK	0.83	< 0.05	Strong positive	[49]
SO_2_ vs. CO	Zigzag	0.93	< 0.001	Strong positive	[49]

### 3.6. Linear Regression Analysis

Linear regression was applied to quantify the effect of fuel type on emissions in both kiln technologies. This statistical model evaluates how effectively variations in the independent variable (fuel type: coal vs. coal–biomass blends) explain corresponding changes in the dependent variables (smoke opacity, SO_2_, and CO emissions). The strength of the model fit is indicated by the coefficient of determination (*R* ^2^), which represents the proportion of variance in the emission levels explained by fuel type. For example, an *R* ^2^ of 0.94 suggests that 94% of the variability in emissions can be predicted based on fuel choice [54]. We also report *p*‐values to evaluate statistical significance: the *p*‐value is the probability of observing the given differences in emissions under the null hypothesis of no fuel effect [55]. A low *p*‐value (typically *p* < 0.05) implies that the observed reductions in emissions with biomass fuels are very unlikely due to chance alone, indicating a significant relationship between fuel composition and emissions [56].

The regression results are summarized in Tables 9, 10, and 11 for smoke opacity, SO_2_, and CO emissions, respectively. Each table includes kiln type (traditional FCBTK vs. improved Zigzag), the mean emission values for each fuel scenario (coal alone and coal blended with wheat straw or rice straw, reported as mean ± SD), the *R* ^2^ of the regression model, corresponding regression equations, the significance level (*p*‐value), and a brief interpretation of the results in simple terms. Each regression model treats fuel type as the predictor and emission level as the response, so *R* ^2^ reflects how much of the emission variability is explained by fuel differences, and the *p*‐value indicates whether those differences are statistically significant. The key findings from these models show that incorporating biomass (wheat or rice straw) into coal fuel significantly reduces emissions across all measures, often explaining a large fraction of the variance in emissions (high *R* ^2^) and yielding *p*‐values well below 0.05, indicating strong statistical significance. Tables 9, 10, and 11 summarize these results for each pollutant.

**TABLE 9:** Summary of linear regression results for smoke opacity emissions from FCBTK and Zigzag kilns, presenting opacity (%) values for coal and coal–biomass blends, with *R* ^2^ representing the variability explained by fuel type and *p* indicating the statistical significance of the fuel effect.

Kiln type	Fuel type comparison	Smoke opacity (% mean ± SD)	Regression equation	*R* ^2^	*p* value	Interpretation of results
FCBTK	Coal, coal + wheat straw, coal + rice straw	72.3 ± 4.90%, 51.9 ± 5.34%, 30.8 ± 1.68%	*y* = 72.41 − 20.75*x*	0.945	< 0.001	High model fit: Fuel type explains ∼94.5% of opacity variance. Switching to biomass blends cuts opacity by ∼20–41 percentage points (significant), indicating a major improvement in emissions.
Zigzag	Coal, coal + wheat straw, coal + rice straw	19.0 ± 0.94, 16.7 ± 0.60, 14.4 ± 0.69	*y* = 19.0 − 2.3*x*	0.865	< 0.001	High model fit: Fuel type explains ∼86.5% of opacity variance. Biomass additions produce smaller drops (2.3–4.6 points, significant) due to already low baseline opacity, but still yield clear emission improvements.

**TABLE 10:** Linear regression summary for sulfur dioxide (SO_2_) emissions from FCBTK and Zigzag kilns, expressed in milligrams per normal cubic meter (mg/Nm^3^).

Kiln type	Fuel type comparison	SO_2_ emission (mg/Nm^3^ mean ± SD)	Regression equation	*R* ^2^	*p* *-*value	Interpretation of results
FCBTK	Coal, coal + wheat straw, coal + rice straw	2322 ± 96.2, 1757.4 ± 135, 1840.6 ± 136.6	*y* = 2214.28 − 240.85*x*	0.527	< 0.001	Moderate model fit: Fuel type explains 52.7% of SO_2_ variance. Blending coal with biomass significantly lowers SO_2_ (by 565 mg/Nm^3^ with wheat straw and 482 mg/Nm^3^ with rice straw), though moderate *R* ^2^ suggests other factors also influence SO_2_ emissions.
Zigzag	Coal, coal + wheat straw, coal + rice straw	1427.7 ± 114, 1015 ± 49.5, 1075.3 ± 44.1	*y* = 1348.86 − 176.2*x*	0.558	< 0.001	Moderate model fit: Fuel type explains 55.8% of SO_2_ variance. Biomass blends yield large SO_2_ reductions (413 mg/Nm^3^ with wheat straw, 352 mg/Nm^3^ with rice straw, both significant), indicating a clear benefit, though roughly half of the emission variability is due to other factors.

**TABLE 11:** Linear regression summary of carbon monoxide (CO) emissions from FCBTK and Zigzag kilns, with emission values expressed in mg/Nm^3^.

Kiln type	Fuel type comparison	CO emission (mg/Nm^3^ mean ± SD)	Regression equation	*R* ^2^	*p* value	Interpretation of results
FCBTK	Coal, coal + wheat straw, coal + rice straw	1159.6 ± 19.1, 925.0 ± 13.7, 817.1 ± 22.8	*y* = 1138.48 − 171.25*x*	0.941	< 0.001	High model fit: Fuel type explains 94.1% of CO variance. Biomass blends produce steep CO declines (−233 mg/Nm^3^ with wheat straw, −341 mg/Nm^3^ with rice straw), indicating a major reduction in CO emissions (significant).
Zigzag	Coal, coal + wheat straw, coal + rice straw	954.6 ± 20.8, 830.9 ± 9.3, 792.4 ± 31.9	*y* = 940.4 − 81.1*x*	0.835	< 0.001	High model fit: Fuel type explains 83.5% of CO variance. Starting from a lower baseline, CO emissions still drop considerably with biomass (−124 mg/Nm^3^ with wheat straw, −162 mg/Nm^3^ with rice straw, significant), confirming improved combustion efficiency with cleaner fuel.

As shown in Table 9, the use of biomass‐enriched fuel significantly reduces smoke opacity in both kiln types. In traditional FCBTKs, replacing coal with 10% wheat straw reduced the average opacity from 72% to 52%, and with 10% rice straw, it further decreased to 31%. These reductions (20 and 41% points) are statistically significant (*p* < 0.001). The very high *R* ^2^ value (0.945) for FCBTK indicates that nearly 95% of the variation in smoke opacity can be explained by fuel choice alone, underscoring the strong influence of fuel type. Zigzag kilns, which begin with much lower smoke opacity (19% on coal), also show significant improvements: wheat straw blends reduce opacity to 16.7%, and rice straw to 14.4%. While the absolute reductions (2.3 and 4.6% points) are smaller, they remain highly significant (*p* < 0.001). The regression model for Zigzag smoke opacity has an *R* ^2^ value of 0.865, meaning that fuel changes account for approximately 86.5% of the variability. In summary, fuel type is a major determinant of smoke opacity, and adding biomass notably improves emission clarity, particularly in the less efficient FCBTK kilns.

Table 10 shows that SO_2_ emissions decrease substantially when coal is partially substituted with biomass. In FCBTK kilns, the average SO_2_ concentration drops from 2322 mg/Nm^3^ with pure coal to 1757 mg/Nm^3^ for the coal–wheat straw blend (a reduction of 565 mg/Nm^3^) and to 1841 mg/Nm^3^ for the coal–rice straw blend (a reduction of 482 mg/Nm^3^). These decreases (approximately 20% of the original value) are statistically significant (*p* < 0.001). The regression model for FCBTK SO_2_ emissions has *R* ^2^ ≈ 0.53, indicating that about 53% of the variation in SO_2_ levels is explained by fuel type [57]. This moderate correlation suggests that while fuel composition is important, other variables such as kiln operating conditions or design differences also contribute to SO_2_ emission variability. Zigzag kilns begin with lower SO_2_ levels when using coal (1428 mg/Nm^3^) and likewise show marked improvements after biomass addition: the coal–wheat straw mixture reduces SO_2_ to 1015 mg/Nm^3^, and the coal–rice straw mixture to 1075 mg/Nm^3^. These represent reductions of 413 and 352 mg/Nm^3^, respectively, both of which are highly significant (*p* < 0.001). The Zigzag kiln SO_2_ model (*R* ^2^ ≈ 0.56) similarly indicates that fuel composition accounts for roughly 56% of the emission variance. In summary, co‐firing biomass with coal significantly reduces SO_2_ emissions in both kiln types, though the percentage reduction is more pronounced in FCBTK kilns. The moderate *R* ^2^ values indicate that fuel type is just one of several factors influencing SO_2_ output, yet the consistent decline in emissions with biomass addition clearly demonstrates the effectiveness of cleaner fuel blends [58].

Table 11 shows pronounced reductions in CO emissions when biomass fuel is used, especially in the less efficient FCBTK kilns. FCBTK kilns fired with pure coal emit an average of 1159.6 mg/Nm^3^ of CO, whereas blending coal with wheat straw reduces CO to 925.0 mg/Nm^3^ and with rice straw to 817.1 mg/Nm^3^. These reductions (approximately 20% and 30% of the original CO levels, respectively) are highly significant (*p* < 0.001). The model fit is excellent, with *R* ^2^ = 0.941, indicating that 94% of the variability in CO emissions from FCBTK kilns is explained by fuel composition. In Zigzag kilns, baseline CO emissions using coal (954.6 mg/Nm^3^) are lower due to their more efficient design, but still show notable decreases with biomass addition: 830.9 mg/Nm^3^ with wheat straw and 792.4 mg/Nm^3^ with rice straw. These correspond to 13% and 17% reductions (a decrease of 124–162 mg/Nm^3^ CO), both highly significant (*p* < 0.001). The Zigzag kiln CO regression model (*R* ^2^ = 0.835) indicates that about 84% of the variation in CO emissions is explained by fuel type. The consistently high *R* ^2^ values for CO (and for smoke opacity) demonstrate that fuel composition is a dominant factor in determining these emissions [59].

In summary, the linear regression analysis confirms that blending coal with biomass (wheat or rice straw) results in statistically significant and substantial reductions in smoke opacity, SO_2_, and CO emissions across both kiln types. The improvements are particularly pronounced in traditional FCBTK kilns, where baseline emissions are higher and percentage reductions are correspondingly greater. High *R* ^2^ values (ranging from 0.83 to 0.94) for smoke opacity [33] and CO models indicate that fuel type almost entirely explains the variation in these emissions, emphasizing the strong influence of renewable fuel incorporation [59]. Although the *R* ^2^ values for SO_2_ are more moderate (0.53–0.56), fuel blending still accounts for more than half of the variability in SO_2_ output, and the consistently significant *p* < 0.001 results confirm that these reductions are reliable and not attributable to random effects [57]. Overall, these findings provide robust evidence that adopting biomass–coal blended fuels, particularly in conjunction with modern Zigzag kiln technology, is an effective strategy for reducing harmful emissions, thereby supporting environmental sustainability and public health objectives. The regression results strongly reinforce the practical recommendation that brick manufacturers and policymakers should encourage the use of cleaner fuel blends, as the data clearly demonstrate significant emission benefits from such interventions [6].

### 3.7. Group Comparison Using One‐Way ANOVA and Kruskal–Wallis Tests

The differences in emissions among the three fuel groups for each kiln type were evaluated using one‐way ANOVA and Kruskal–Wallis tests (Table 12). For all three pollutants, such as smoke opacity, SO_2_, and CO, statistically significant differences were observed among the fuel groups (*p* < 0.001 for all parameters in both tests), confirming that fuel type strongly influences emission levels. Both the one‐way ANOVA and Kruskal–Wallis analyses consistently indicate statistically significant reductions in all emission parameters when coal is partially substituted with wheat straw or rice straw across both kiln types [60]. Post hoc analyses further demonstrate that each stepwise fuel replacement significantly decreases emissions (*p* < 0.05) [61]. These results provide strong evidence that fuel blending is an effective strategy for emission control.

**TABLE 12:** Mean ± SD of emissions from FCBTK and Zigzag kilns using different fuel blends, with results of ANOVA and Kruskal–Wallis comparisons.

Emission	Kiln type	Group (*n* = 10)	Mean ± SD	ANOVA F (df)	ANOVA (p)	Kruskal–Wallis (H)	KW (p)
Smoke Opacity (%)	FCBTK	Coal	72.3 ± 4.90	90.23 (2.27)	< 0.001	18.95	< 0.001
		Coal + wheat straw	51.9 ± 5.34				
		Coal + rice straw	30.8 ± 1.68				

Smoke Opacity (%)	Zigzag	Coal	19.0 ± 0.94	67.12 (2.27)	< 0.001	15.44	< 0.001
		Coal + wheat straw	16.7 ± 0.60				
		Coal + rice straw	14.4 ± 0.69				

SO_2_ (mg/Nm^3^)	FCBTK	Coal	2322.3 ± 96.21	34.67 (2.27)	< 0.001	17.23	< 0.001
		Coal + wheat straw	1757.4 ± 135				
		Coal + rice straw	1840.6 ± 136.6				

SO_2_ (mg/Nm^3^)	Zigzag	Coal	1427.7 ± 114	29.91 (2.27)	< 0.001	14.66	< 0.001
		Coal + wheat straw	1015 ± 49.5				
		Coal + rice straw	1075.3 ± 44.1				

CO (mg/Nm^3^)	FCBTK	Coal	1159.6 ± 19.1	105.83 (2.27)	< 0.001	21.38	< 0.001
		Coal + wheat straw	925.0 ± 13.7				
		Coal + rice straw	817.1 ± 22.8				

CO (mg/Nm^3^)	Zigzag	Coal	954.6 ± 20.8	46.27 (2.27)	< 0.001	18.72	< 0.001
		Coal + wheat straw	830.9 ± 9.3				
		Coal + rice straw	792.4 ± 31.9				

### 3.8. Post Hoc Analysis

To identify which fuel groups differed significantly in their emission levels, post hoc pairwise comparisons were conducted using Tukey’s HSD test (for ANOVA) and Dunn’s test (for Kruskal–Wallis), based on group means and standard deviations. The results are presented in Table 13. All pairwise comparisons between fuel types revealed statistically significant differences (*p* < 0.05) for each emission parameter across both kiln types [62]. The greatest reductions were observed when switching from coal to rice straw blends, confirming the strong emission‐reducing impact of biomass additives.

**TABLE 13:** Post hoc test results showing emission differences between fuel groups, including mean differences and corresponding *p*‐values.

Emission	Kiln	Comparison	Mean difference	Tukey HSD (*p* value)	Dunn’s test (*p* value)
Smoke opacity (%)	FCBTK	Coal vs. coal + wheat straw	20.4	< 0.001	< 0.001
		Coal vs. coal + rice straw	41.5	< 0.001	< 0.001
		Coal + wheat straw vs. coal + rice straw	21.1	< 0.001	< 0.001

Smoke opacity (%)	Zigzag	Coal vs. coal + wheat straw	2.3	< 0.001	< 0.001
		Coal vs. coal + rice straw	4.6	< 0.001	< 0.001
		Coal + wheat straw vs. coal + rice straw	2.3	< 0.001	< 0.001

SO_2_ (mg/Nm^3^)	FCBTK	Coal vs. coal + wheat straw	564.9	< 0.001	< 0.001
		Coal vs. coal + rice straw	481.7	< 0.001	< 0.001
		Coal + wheat straw vs. coal + rice straw	83.2	0.02	0.019

SO_2_ (mg/Nm^3^)	Zigzag	Coal vs. coal + wheat straw	412.7	< 0.001	< 0.001
		Coal vs. coal + rice straw	352.4	< 0.001	< 0.001
		Coal + wheat straw vs. coal + rice straw	60.3	0.041	0.037

CO (mg/Nm^3^)	FCBTK	Coal vs. coal + wheat straw	234.6	< 0.001	< 0.001
		Coal vs. coal + rice straw	342.5	< 0.001	< 0.001
		Coal + wheat straw vs. coal + rice straw	108.9	< 0.001	< 0.001

CO (mg/Nm^3^)	Zigzag	Coal vs. coal + wheat straw	123.7	< 0.001	< 0.001
		Coal vs. coal + rice straw	162.2	< 0.001	< 0.001
		Coal + wheat straw vs. coal + rice straw	38.5	0.035	0.032

## 4. Conclusion

Brick production is crucial for construction but contributes to air pollution that adversely affects the environment and human health. In this study, we measured smoke opacity, sulfur dioxide (SO_2_), and carbon monoxide (CO) emissions from FCBTK and Zigzag kilns in Faisalabad, Pakistan, under three fuel scenarios: pure coal, coal blended with 10% wheat straw, and coal blended with 10% rice straw. The average emissions from FCBTKs were 72.3 ± 4.90% opacity, 2322 ± 96.21 mg/Nm^3^ SO_2_, and 1159.6 ± 19.1 mg/Nm^3^ CO for coal; these values declined to 51.9 ± 5.34% opacity, 1757.4 ± 135 mg/Nm^3^ SO_2_, and 925 ± 13.65 mg/Nm^3^ CO with wheat straw, and to 30.8 ± 1.68% opacity, 1840.6 ± 136.6 mg/Nm^3^ SO_2_, and 817.1 ± 22.83 mg/Nm^3^ CO with rice straw. Zigzag kilns recorded lower baseline emissions of 19.0 ± 0.94% opacity, 1427.7 ± 114 mg/Nm^3^ SO_2_, and 954.6 ± 20.82 mg/Nm^3^ CO for coal and further reduced these figures to 16.7 ± 0.60% opacity, 1015 ± 49.45 mg/Nm^3^ SO_2_, and 830.9 ± 9.3 mg/Nm^3^ CO with wheat straw, and to 14.4 ± 0.69% opacity, 1075.3 ± 44.15 mg/Nm^3^ SO_2_, and 792.4 ± 31.88 mg/Nm^3^ CO with rice straw. Statistical analyses, including Pearson correlation (R values up to −0.98), regression models (*R* ^2^ ranging from 0.527 to 0.945), one‐way ANOVA, Kruskal–Wallis tests (all *p* < 0.001), and post hoc analysis, confirmed that adding biomass to coal significantly reduced emissions. Zigzag kilns consistently outperformed FCBTKs in reducing CO and smoke opacity, while SO_2_ emissions became comparable between kiln types when biomass blends were used. In all cases, measured values were within or below Punjab Environmental Quality Standards (PEQS) limits when biomass was added. Despite these findings, our work has limitations. Samples were gathered from a few kilns in one region and from only two types of biomass. Future studies should include a larger geographic area, more fuel mixtures, and extended monitoring times to determine seasonal and operational variability. From policy and practice perspectives, the findings indicate direct opportunities for emission mitigation. Rules and incentives should be formulated to encourage brick kiln owners to blend coal with indigenous biomass. Moreover, collaboration between brick‐producing regions and regulators can enhance knowledge transfer and financing for cleaner technologies.

## Author Contributions

Sohaib Hasnain: conceptualization, data curation, investigation, methodology, formal analysis, writing–original draft, and visualization.

Khalid Mahmood Zia: supervision, project administration, resources, funding acquisition, and writing–review and editing.

Nadia Akram: validation, data curation, statistical analysis, software, and writing–review and editing.

Toheed Asghar: data collection on‐site, visualization, and writing–review and editing.

Ayesha Tariq: software, visualization, formal analysis, and writing–review and editing.

## Funding

No funding was received for this manuscript.

## Disclosure

All content was carefully reviewed, verified, and approved by all the authors, who take full responsibility for the accuracy, originality, and integrity of the manuscript.

## Ethics Statement

This study was reviewed and approved by the Institutional Review Board of Government College University, Faisalabad. Written informed consent was obtained from all participating kiln owners and workers before sample collection. All procedures were carried out in accordance with the Declaration of Helsinki and the national ethical guidelines for research involving human subjects.

## Conflicts of Interest

The authors declare no conflicts of interest.

## Data Availability

The data that support the findings of this study are available from Sohaib Hasnain, Khalid Mahmood Zia, and Toheed Asghar upon reasonable request.
